# Drug Conjugates for Targeting Eph Receptors in Glioblastoma

**DOI:** 10.3390/ph13040077

**Published:** 2020-04-23

**Authors:** Puja Sharma, Callie Roberts, Denise Herpai, Izabela D. Fokt, Waldemar Priebe, Waldemar Debinski

**Affiliations:** 1 Brain Tumor Center of Excellence, Wake Forest Baptist Medical Center Comprehensive Cancer Center, Winston-Salem, NC 27157, USA; psharma@wakehealth.edu (P.S.); callieroberts@rocketmail.com (C.R.); dgibo@wakehealth.edu (D.H.); 2 Department of Experimental Therapeutics, Division of Cancer Medicine, MD Anderson Cancer Center, Houston, TX 77054, USA; ifokt@mdanderson.org (I.D.F.); wpriebe@me.com (W.P.)

**Keywords:** Eph receptors, multiple-receptor targeting, glioblastoma, ligand–drug conjugates, doxorubicin

## Abstract

Glioblastoma (GBM) is a complex and heterogeneous tumor that warrants a comprehensive therapeutic approach for treatment. Tumor-associated antigens offer an opportunity to selectively target various components of the GBM microenvironment while sparing the normal cells within the central nervous system. In this study, we conjugated a multivalent vector protein, QUAD 3.0, that can target four receptors: EphA3, EphA2, EphB2, and also IL-13RA2, spanning virtually 100% of the GBM microenvironment, to doxorubicin derivatives. The conjugates effectively bound to all four receptors, although to varying degrees, and delivered cytotoxic loads to both established and patient-derived GBM cell lines, with IC_50_ values in the low nM range. The conjugates were also non-toxic to animals. We anticipate that the QUAD 3.0 Dox conjugates will be further used in preclinical models and possibly clinics in the foreseeable future.

## 1. Introduction

Glioblastoma (GBM) is a stage IV high-grade glioma, a brain tumor that is believed to arise from the glial cells of the central nervous system (CNS) [1]. It accounts for 56% of gliomas, with over 12,000 new cases being diagnosed every year in the US [2]. The standard of care for GBM includes surgery, chemotherapy, radiation, and tumor-treating fields (TTFs). Even with aggressive treatment regimens, the median survival of GBM patients is only 15–17 months as of now [3]. Some of the challenges in treating GBM include the molecular and genetic heterogeneity [4], infiltrative nature of the tumor cells, location of the tumor, resistance to therapy, and tumor neovasculature [5,6,7]. These challenges warrant new therapeutic approaches that can address these issues.

The advent of tumor-associated antigens (TAAs) has introduced an array of targeted therapies for GBM [8,9]. TAAs are lacking in normal cells of the CNS, which opens a window for selective targeting of GBM cells while sparing the normal cells in the tumor microenvironment. Some of the TAAs in GBM include IL-13RA2, Survivin, Wilms tumor 1, EphA2, EphA3, EphB2, melanoma-associated antigen 1 (MAGE-1), glycoprotein 100 (gp100), epidermal growth factor receptor (HER-2), tyrosine-related protein 2 (TRP-2), epidermal growth factor receptor variant III (EGFRvIII), transferrin, α_v_β_3_ and α_v_β_5_ integrins, c-Met receptor, transforming growth factor receptor (TGFR), platelet-derived growth factor receptor (PDGFR), vascular endothelial growth factor receptor (VEGFR), folate receptor, and lactoferrin receptor [10]. Some of the TAAs include plasma membrane receptors that are selectively expressed in tumor cells and its microenvironment but not in their normal counterparts. The targeting of such receptors has been achieved in different ways. These include the use of ligand- [11,12,13], antibody- [14,15], and liposome/nanoparticles-based therapeutic agents [16,17]. Additionally, immunotherapy has utilized antigen-pulsed dendritic cells [18], chimeric antigen receptor T-cells (CAR T-cells) [19,20,21,22], and vaccines [23,24,25] to selectively target TAA in tumors. While immunotherapy has yet to show clinical benefit in GBM, it is considered a fourth pillar of cancer treatment and holds much promise [26].

The Eph receptors represent the largest family of receptor tyrosine kinases. It comprises nine EphA receptors that bind to five ephrinA ligands, and five EphB receptors that bind to three ephrinB ligands. Receptors EphB2 and EphA4 can bind to ephrin ligands of different classes [27]. Their implications have been discovered in various developmental [28,29], physiological [30,31,32], and pathological phenomena [33,34,35]. Moreover, increasing indications of their involvement in tumor invasion, initiation [36], tumor immunity [37], and tumor angiogenesis [12,35] have been observed. Specifically, EphA2 and EphA3 have been shown to be expressed individually in 60% of GBM patients, including in regions of tumor neovasculature, tumor-associated immune cells, and tumor-infiltrating cells [12,35,38]. The increased expression of EphA2 and EphA3 in GBM patients is associated with poor patient prognosis and survival [39,40]. Similarly, the expression of EphB2 has been coupled with increased migration and invasion of GBM cells [41,42].

The Eph receptors have been targeted using antibodies, kinase inhibitors, radionucides, and peptides that mimic the receptor ligand and carry a cytotoxic load [10]. The Eph receptors internalize once bound to their ligands and resurface later [12,13]. This allows ligand-based or ligand-mimicking peptide-based therapeutics to be administered in multiple doses given the re-expression of the receptors over time. Recently, an ephrinA5-based cytotoxin that can simultaneously target EphA3, EphA2, and EphB2 receptors showed cytotoxic effects in GBM cells [12]. Another recent completion of a phase I clinical trial targeting IL-13RA2 and EphA2 in spontaneous canine gliomas has shown promising results. The cytotoxic cocktail was able to decrease the tumor volume by up to 97%, increase the overall survival of canine patients, and improve the quality of life [43]. IL-13RA2 is a GBM TAA that has been shown to be overexpressed in up to 75% of GBM patients [44]. Since its discovery, IL-13RA2 has been used as a target for GBM in more than 30 different experimental, preclinical, and clinical therapies, making it one of the most well-studied and promising targets for GBM [10,11,17,18,23,43,44,45,46,47,48,49,50,51,52,53,54,55]. Studies have demonstrated that the overexpression of IL-13RA2 correlates with a higher GBM grade and poor patient survival [56]. Furthermore, IL-13RA2 is expressed in the mesenchymal-type tumor cells within the GBM microenvironment [57,58]. Targeting IL-13RA2 alone showed therapeutic benefit by rendering the tumor as less invasive [59].

Antibody–drug conjugates (ADCs) are a promising class of therapeutics that can selectively target TAAs while sparing the normal cells of the tumor microenvironment. Since the approval of the first antibody–drug conjugate (ADC) by the FDA in 2000 more than 80 ADCs have been developed clinically [60]. Until 2017, four ADCs were approved by the FDA for the treatment of various types of cancers. Namely, Mylotarg, which targets CD33 for acute myeloid leukemia [61]; Adcetris, which targets CD30 for Hodgkin’s lymphoma [62]; Kadcyla, which targets HER2-positive breast cancer [63]; and Besponsa, which targets CD22 for acute lymphoblastic leukemia [64,65]. Recently, the FDA has approved TR1801-ADC(MT_8633) from Tanabe Research Labs that targets cMet-positive solid tumors [66]. This investigational new drug (IND) is conjugated with a potent pyrrolobenzodiazepine dimer (PBD) toxin that intercalates the DNA and inhibits cell replication, eventually causing cell death. Another ADC, Polivy, developed by Roche and Seattle Genetics, was approved by the FDA for the treatment of diffuse large B-cell lymphoma (DLBCL) [67]. Polivy targets CD79b, which is primarily expressed in the B-cells of the tumor. Similarly, ICON-2 was developed by Iconic Therapeutics, targets tissue factor (also known as CD142), and is conjugated to an anti-mitotic agent momomethyl auristatin E (MMAE). Its efficacy has been well demonstrated in a variety of solid tumors [68,69]. DS-8201 [70], DREAMM-2 [71], Enfortumab-Vedotin [72,73], and Sacituzumab Govitecan [74,75] are ADCs that are emerging as promising therapeutics for various types of cancers [76]. It is evident from clinical and preclinical results from the past few decades that ADCs are a reliable and promising line of therapeutics for a myriad of different cancers, including GBM.

In this study, we utilized a multivalent vector protein, QUAD 3.0, that contains ephrinA5 (eA5) and IL-13.E13K, which concurrently target EphA3, EphA2, EphB2, and IL-13RA2 receptors [46]. eA5 binds to the receptors EphA3, EphA2, and EphB2 [12]. IL-13.E13K is a modified version of the IL-13 ligand that selectively binds to the GBM-specific IL-13RA2 and not to the physiological IL-4RA/IL-13RA1 receptor complex [77,78]. In targeting these four receptors at once, we anticipated that the following would be achieved: (a) Therapeutic coverage of almost 100% of the tumor and its heterogeneous microenvironment; (b) multiple targeting that reduces the chances of antigen loss, allowing significantly less room for therapeutic resistance; and (c) targeting of the mesenchymal subtype along with tumor-associated immune, vascular, infiltrative, and invasive cells within the tumor microenvironment, rendering the tumor less aggressive after therapy. Here, we successfully conjugated QUAD 3.0 to doxorubicin (Dox) variants. In particular, QUAD 3.0 was conjugated to Dox variants WP936, WP1737, and WP1244. WP936 was cytotoxic to MES-SA uterine sarcoma cells in vitro, with an IC_50_ value of 130 nM. WP1737 is a thiol-reactive derivative of Berubicin. Berubicin was cytotoxic to multiple myeloma cells U266, MM1S, and ARP-1 in vitro, with IC_50_ values of 5.99, 5.21, and 3.99 nM, respectively. Berubicin has been shown to be able to cross the blood–brain-barrier (BBB). Among the three variants, WP1244 was the most potent. It was cytotoxic to U87 and D54 glioma cells, Colo357-FG metastatic pancreatic adenocarcinoma cells, and H441 papillary adenocarcinoma cells, with IC_50_ values of 0.2, 1.08, 3.1, and 0.91 nM respectively [11,79,80,81,82,83,84]. Our results show that the QUAD 3.0-Dox conjugates were cytotoxic to established and patient-derived GBM cell lines in vitro, with IC_50_ values in the low nM range.

## 2. Results

### 2.1. QUAD 3.0 Was Successfully Conjugated to Dox Derivatives

**Figure 1:**
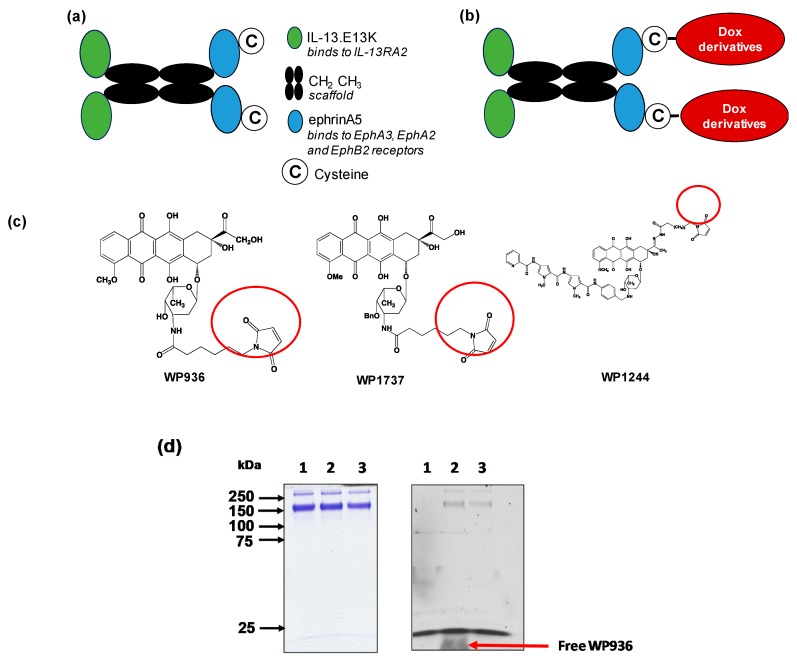
Dox derivatives conjugation to QUAD 3.0. (**a**) A schematic of the QUAD 3.0 protein showing the CH_2_ CH_3_ domains of the human IgG1 used as a scaffold. IL-13.E13K, a modified version of the IL-13 ligand, is present in the N-terminal of the molecule and ephrinA5 along with a cysteine present in the C-terminal end of the molecule; (**b**) A schematic of the conjugation of Dox derivatives to the QUAD 3.0 molecule; (**c**) The structure of the three Dox derivatives used in the study that are thiol reactive (circled) and form a stable thioether bond with the thiol present in the cysteine residue; (**d**) SDS-PAGE (left panel) and corresponding fluorescent image the same gel (right panel) of QUAD 3.0 (lane 1) and its QUAD 3.0-WP936 conjugate before (lane 2) and after the removal of excess unconjugated WP936 using a Zeba desalting column (lane 3). To detect the fluorescently labeled protein, the gel was scanned using an Amersham AI 600RGB digital scanner.

QUAD 3.0 is a multivalent vector protein containing IL-13.E13K and eA5 in the N-terminal and C-terminal ends of the molecule, respectively [46,85]. The protein was designed with CH2-CH3 domains of the human IgG1 as a scaffold that holds the IL-13.E13K and ephrinA5 proteins together (Figure 1a). Additionally, QUAD 3.0 contains a cysteine at the C-terminal end of the molecule. This provides the molecule with a reactive thiol at the C-terminal end, allowing it to be conjugated to other proteins and chemotherapeutic agents without chemical modifications. This design of avoiding chemical modification prevents manipulation of the protein, which could have potentially interfered with the binding ability of the protein to the EphA3, EphA2, EphB2, and IL-13RA2 receptors.

We successfully conjugated the QUAD 3.0 to three variants of doxorubicin: WP936 [11,79,80], WP1737, and WP1244 [81] (Figure 1c); these variants are designed to be thiol reactive. Berubicin is the first powerful anthracycline known to cross the BBB and sequester primarily in the tumor tissue [82,83]. We designed the conjugates to not interfere with the IL-13.E13K and ephrinA5 regions of the QUAD 3.0, which are responsible for effective binding to the receptors.

While the molecular weights of the Dox variants range from 737 to 981 g/mol [81,86], QUAD 3.0 homodimer has a molecular weight of 120 kD. Hence, we did not see a shift in the band size of the QUAD 3.0-WP936 on an SDS-PAGE (Figure 1d, left panel). However, the Dox derivatives are fluorescent. Images taken at 570 nm demonstrated fluorescent bands that correspond to the QUAD 3.0-Dox conjugate; unconjugated Dox derivatives were effectively removed using a desalting column (e.g., Figure 1d, right panel). The estimated drug to vector ratio (DAR) for QUAD 3.0-WP936 (the measure of drug conjugation to ligand efficiency) was 0.41 WP936 per QUAD. It appears that we still have room to improve the conjugation conditions, which may translate into even more active conjugates.

### 2.2. QUAD 3.0-Dox Derivative Conjugates Bind to EphA3, EphA2, EphB2, and IL-13RA2 Receptors

**Figure 2:**
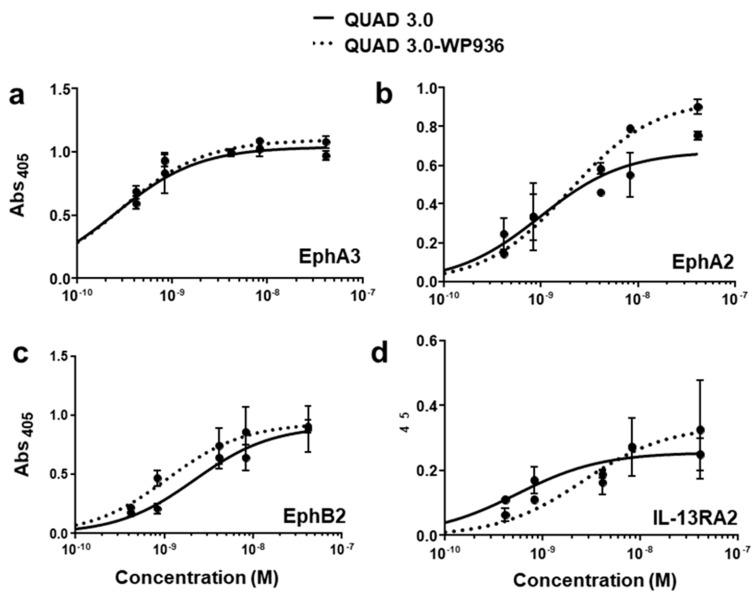
QUAD 3.0-WP936 binding to EphA3, EphA2, EphB2, and IL-13RA2 receptors. ELISA assay showing the binding of unconjugated QUAD 3.0 and QUAD 3.0-WP936 conjugate to (**a**) EphA3; (**b**) EphA2; (**c**) EphB2; and (**d**) IL-13RA2 receptor proteins.

**Figure 3:**
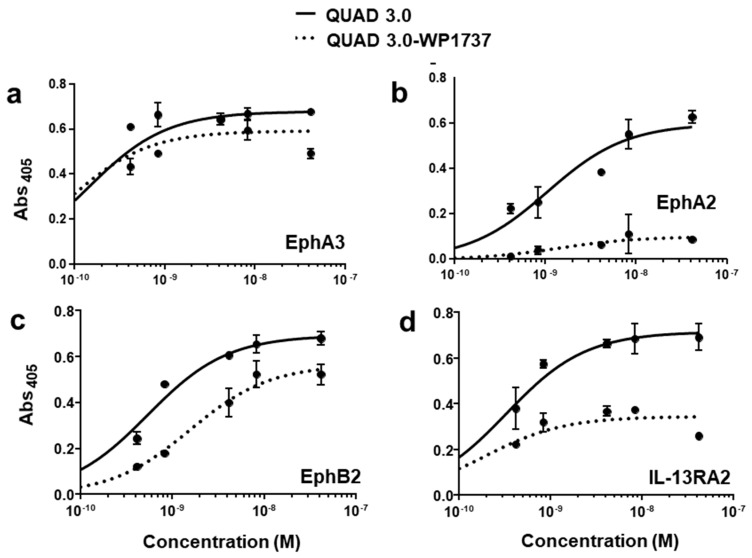
QUAD 3.0-WP1737 binding to EphA3, EphA2, EphB2, and IL-13RA2 receptors. ELISA assay showing the binding of unconjugated QUAD 3.0 and QUAD 3.0-WP1737 conjugate to (**a**) EphA3; (**b**) EphA2; (**c**) EphB2; and (**d**) IL-13RA2 receptor proteins.

**Figure 4:**
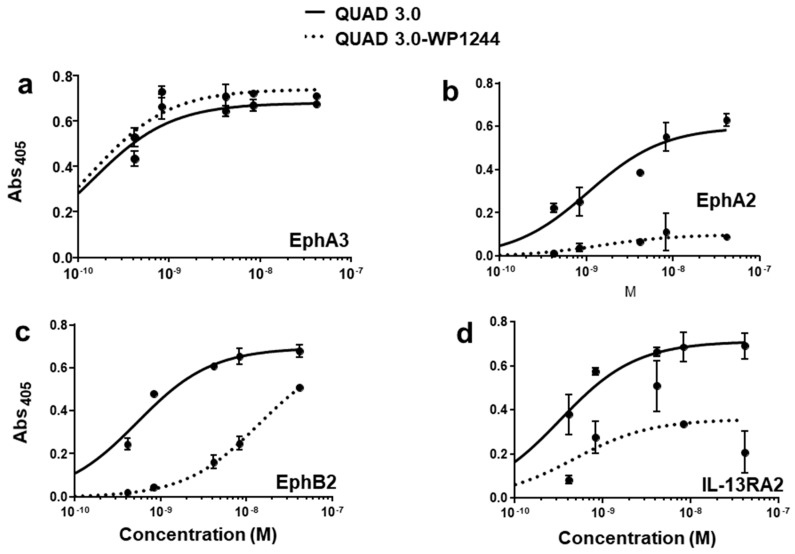
QUAD 3.0 Dox 1244 binding to EphA3, EphA2, EphB2, and IL-13RA2 receptors. ELISA results showing the binding of unconjugated QUAD 3.0 and QUAD 3.0-WP1244 conjugate to (**a**) EphA3; (**b**) EphA2; (**c**) EphB2; and (**d**) IL-13RA2 receptor proteins.

QUAD 3.0 was designed to bind EphA3, EphA2, EphB2, and IL-13RA2 receptors expressed in the GBM cells and its microenvironment. After successful conjugation with the Dox derivatives, we wanted to test if the QUAD 3.0-Dox derivatives still effectively bound to the receptors. We performed ELISA to test the binding ability of unconjugated QUAD 3.0 and QUAD 3.0 conjugated with Dox derivatives. The unconjugated QUAD 3.0 and conjugated QUAD 3.0-WP936 bound very efficiently to the receptors EphA3 (Figure 2a), EphA2 (Figure 2b), and EphB2 (Figure 2c), and IL-13RA2 (Figure 2d). There was a change in the Kd of binding to the IL-13RA2 (Table 1), but the Bmax values remained very similar. While the QUAD 3.0-WP1737 conjugate still bound with similar Kds to EphA3 (Figure 3a), EphA2 (Figure 3b), and EphB2 (Figure 3c), and IL-13RA2 (Figure 3d) receptors, the Bmax values diminished, especially for the binding with the EphA2 and IL-13RA2. QUAD 3.0-WP1244 conjugate also bound with similar Kds to EphA3 (Figure 4a), EphA2 (Figure 4b), and IL-13RA2 (Figure 4d), and its binding to the EphB2 receptor was significantly impaired (Figure 4c). Moreover, the Bmax values decreased, especially for the EphA2 and IL-13RA2 receptors. The Kd values of both the unconjugated QUAD 3.0 and the UAD 3.0-Dox conjugates were in the picomolar to nanomolar range (Table 1). These results demonstrate that conjugating the QUAD 3.0 with Dox derivatives variably affects the ability of the protein to bind to the targeted receptors, with QUAD 3.0-WP936 retaining most of the binding characteristics of the QUAD 3.0 protein.

### 2.3. QUAD 3.0-WP936 Conjugate Binds and Is Internalized by the GBM Cells

**Figure 5:**
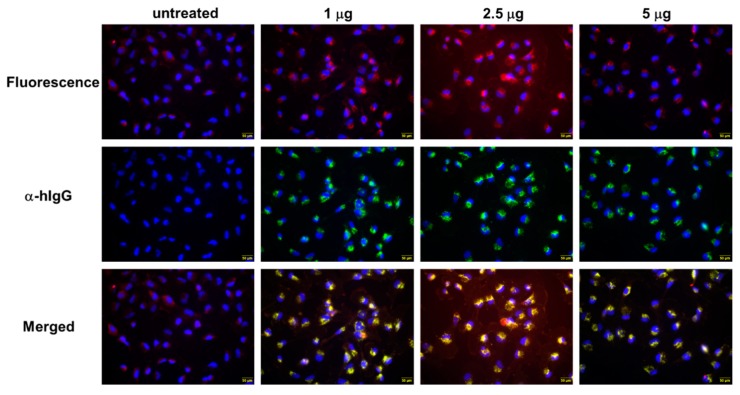
QUAD 3.0-WP936 binding and internalization to U-251 GBM cells. The cells were treated with 1.0–5.0 μg of the conjugate for 4 h. WP936 was detected by red fluorescence in a TRITC channel and QUAD 3.0 by green fluorescence through Alexa-488-conjugated anti-human IgG. Sections were counterstained for DAPI (blue fluorescence) (scale bar: 50 μm).

To test whether the QUAD 3.0-WP936 conjugate can bind live cells and be internalized in order to deliver toxic cargo, we treated the U-251 GBM cells with different amounts of the conjugate for 4 h. As expected, the avid internalization of QUAD 3.0-WP936 was observed, and the cells exhibited readily detectable signals for the two individual components of the conjugate: QUAD 3.0 and WP936, a fluorescent derivative of Dox (Figure 5). Thus, the QUAD 3.0-WP936 not only efficiently binds the targeted receptors in vitro but also recognizes live cells and is internalized by them.

### 2.4. QUAD 3.0-Dox Conjugates Are Cytotoxic to Established and Patient-Derived GBM Cells

After effectively conjugating the Dox derivatives to QUAD 3.0 while maintaining at least some of the abilities to bind to the targeted receptors, we wanted to investigate the cytotoxic activities of the conjugates. In order to do so, we used established (U-251 and T98G) and patient-derived (BTCOE 4795) GBM cell lines.

**Figure 6:**
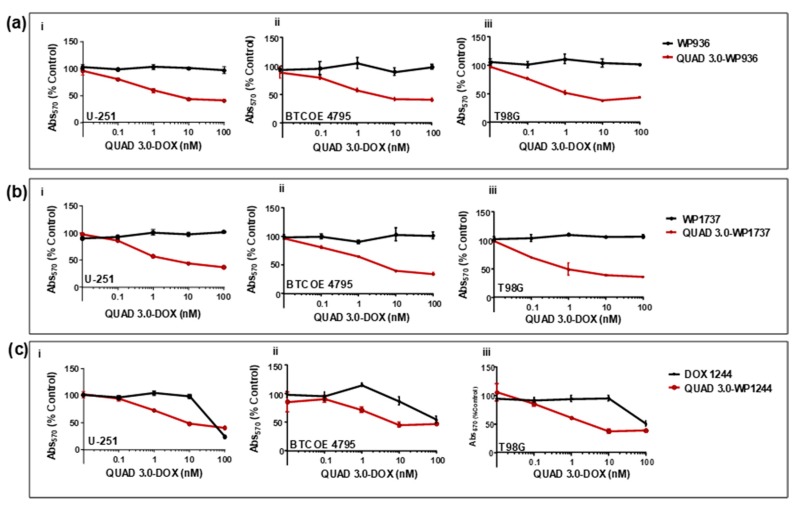
QUAD 3.0-Dox conjugates are cytotoxic to established and patient-derived GBM cell lines. MTT assay of (**a**) unconjugated WP936 and QUAD 3.0-WP936 conjugates in (**i**) U-251, (**ii**) BTCOE 4795, and (**iii**) T98G cells; (**b**) unconjugated WP1737 and QUAD 3.0-WP1737 conjugates in (**i**) U-251, (**ii**) BTCOE 4795, and (**iii**) T98G cells; and (**c**) unconjugated WP1244 and QUAD 3.0-WP1244 conjugates in (**i**) U-251, (**ii**) BTCOE 4795, and (**iii**) T98G cells.

We found that unconjugated WP936 did not show cytotoxic activity in U-251 (Figure 6(ai)); express IL-13RA2, EphA2, EphA3, and little EphB2), BTCOE 4795 (Figure 6(aii)); express IL-13RA2, EphA2, and no EphB2), and T98G (Figure 6(aiii)); express EphA2, but not IL-13RA2 or EphB2) ([12] and data not shown) GBM cells when the cells were exposed to concentrations of up to 100 nM of the drug. However, the QUAD 3.0-WP936 conjugate demonstrated cytotoxic activity in both the established and patient-derived GBM cells (Figure 6(ai–aiii) and Table 2), with IC_50_ values between 1.1 and 3.1 nM. Thus, by conjugating WP936 to the QUAD 3.0, its cytotoxic potential was increased while targeting the GBM receptors of interest.

Similarly, unconjugated WP1737 alone was not able to instill cytotoxic effects in U-251 (Figure 6(bi)), BTCOE 4795 (Figure 6(bii)), and T98G (Figure 6(biii)) when exposed to concentrations up to 100 nM. The IC_50_ values of the QUAD 3.0-WP1737 conjugates ranged from 0.87 to 3.7 nM (Table 2) for these cell lines. Comparable to the WP936 conjugates, these results demonstrate that by conjugating the WP1737 to the QUAD 3.0, the cytotoxicity of the Dox derivative is enhanced in GBM cells.

Unlike the unconjugated WP936 and WP1737, unconjugated WP1244 was cytotoxic to U-251, BTCOE 4795, and T98G at concentrations greater than 10 nM (Figure 6(ci–ciii)). The QUAD 3.0-WP1244 was also cytotoxic to these cells (Figure 6(ci–ciii)). While unconjugated WP1244 was more potent than the unconjugated WP936 and WP1737, the IC_50_ values of the QUAD 3.0-WP1244 conjugate were higher than those of the conjugated counterparts of WP936 and WP1737 and ranged from 1.9 to 7.8 nM (Table 2). These results demonstrate that by conjugating WP1244 to QUAD 3.0, the cytotoxic potential can still be significantly enhanced while targeting GBM cells, but this enhancement is comparatively less perhaps because the WP1244 conjugate demonstrated more impaired binding to the targeted receptors (Figure 4).

### 2.5. Intracranial Injections of QUAD 3.0-WP 936 Conjugate Are Safe in Mice

**Figure 7:**
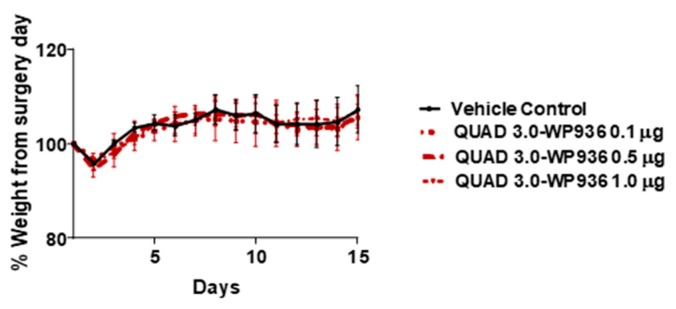
QUAD 3.0-WPD936 is safe in mice. Change in weight (%) of C57BL/6 mice after intracranial injections of QUAD 3.0-WP936.

The cell viability assay results showed that QUAD 3.0-Dox conjugates are cytotoxic to GBM cells, with IC_50_ values between 0.87 and 7.8 nM, while unconjugated WP936 and WP1737 are not cytotoxic at concentrations up to 100 nM. Hence, we used QUAD 3.0-WP936 conjugate to test for toxicity in mouse models. Intracranial injections of QUAD 3.0-WP936 in C57BL/6 mice at different concentrations, 0.1, 0.5, and 1.0 μg of the conjugate in 5 μL (167 nM, 833 nM and 1.7 μM, respectively), did not show impairment in their grooming activities and movement or significant changes in weight (Figure 7). Thus, the QUAD 3.0-WP936 was rendered without gross neurological toxicity in C57BL/6 mice when up to 1.0 μg of the conjugate was administrated intracranially. The toxicity of the conjugate will be studied in larger animal models and with repeated/continuous infusions as well.

## 3. Discussion

GBM is a highly complex and heterogeneous tumor [4]. It is composed of tumor cells, cells infiltrating the tumor microenvironment, tumor-associated immune cells, cells of the tumor neovasculature, and tumor-initiating cells [87,88,89] These variable and dynamic facets of the GBM microenvironment allow the tumor to initiate, grow, sustain, and eventually develop resistance to therapies. Essentially, the GBM microenvironment can be described as a dynamic tumor ecosystem [88,89,90,91]. It is evident that by targeting a single component within this complex GBM microenvironment, desired therapeutic effects cannot be achieved. Treatment options like surgery, chemo, and radiation therapy alone cannot comprehensively target multiple aspects of a GBM microenvironment. Effective treatment modalities for GBM should therefore focus on targeting multiple components of the GBM microenvironment to achieve better prognosis [7,91]. Utilizing TAAs to selectively target GBM offers an improvement over the conventional methods of treatment. However, targeting a single TAA in GBM leads to antigen loss, making the therapy ineffective over time [20,92,93]. We have been advocating for some time that the use of multiple targets in GBM is an excellent strategy; it avoids antigen loss and promises more optimal and comprehensive therapeutic benefits to patients [7].

The targeting of multiple receptors in GBM has shown great promise in experimental and preclinical settings [12,46,94]. The overexpression of Eph receptors in GBM has been well documented [95,96,97]. Particularly, EphA2 overexpression has been observed in vascular regions of GBM, indicating its role in tumor neovascularization [38]. The overexpression of EphA2 mRNA was also inversely related to GBM patient survival [98]. EphA2 overexpression has also been recorded in tumor-initiating cells within the GBM microenvironment, which are known to drive therapy resistance [99]. The EphA3 receptor, while more recently recognized to be overexpressed in GBM, is an equally excellent target. Its expression has been reported in tumor cells, cells that infiltrate the immediate microenvironment, and tumor-initiating cells [12,100]. EphB2 overexpression in GBM has been reported to drive invasion and migration of the tumor cells via the focal adhesion kinase (FAK) pathway [42]. It is imperative that by targeting the EphA3, EphA2, and EphB2 receptors at once, we will be targeting not just the tumor cells, but also key components within the GBM microenvironment that allow GBM to sustain and grow. IL-13RA2 is an additional GBM target that has been shown to be extremely effective in targeting GBM. The combined expression of EphA3, EphA2, EphB2, and IL-13RA2 spans almost 100% of the GBM microenvironment. We aimed to use QUAD 3.0 conjugated with Dox variants to target the GBM microenvironment and successfully demonstrated the effect of the QUAD 3.0-Dox conjugates in killing GBM cells. GBM cells are known to be resistant to chemotherapy and require higher concentrations of drugs, including Dox (e.g., Biomater Sci. 2019, 7, 2102–2122; PLoS ONE. 2014, 9, e103736.).

In summary, we have shown that a multivalent vector protein QUAD 3.0 that targets EphA3, EphA2, EphB2, and IL-13RA2 can be conjugated to Dox derivatives. Our data shows that conjugating Dox derivatives to the QUAD 3.0 variably changed the binding to the four receptors, with the QUAD 3.0-WP936 conjugate being the least affected by conjugating the Dox derivatives with the QUAD 3.0, thus we made the therapeutic agent selective to glioma cells. We observed that the QUAD 3.0-Dox conjugates were cytotoxic in the nM range unlike their unconjugated counterparts when treating GBM cells. Thus, conjugating the Dox derivatives with QUAD 3.0 made the therapeutic agents more potent. The antitumor effects of the conjugates were evident in both established and patient-derived GBM cell lines However, the DAR value for the conjugate can likely be improved, which could translate in a further increase in the conjugate’s antitumor activity. A more detailed mechanism of the conjugates’ action and the extent of the therapeutic window will be further examined in, e.g., receptor knockdown or antireceptor and/or antiligand antibody experiments. This will help to delineate the contribution of the respective receptors to the action of the conjugates and model the future design of this type of targeted drug candidate. The QUAD 3.0 Dox conjugates will be tested in pre-clinical settings like in canine spontaneous tumors in the near future, the most faithful models of human disease.

## 4. Materials and Methods

### 4.1. Cell Lines and Reagents

U-251 and T-98G cells were obtained from American Type Culture Collection (ATCC, Manassas, VA, USA) and cultured as recommended. BTCOE 4795 were isolated from a GBM patient within 20 min post resection and cultured as described previously [12]. They were authenticated back to the original patient tumor by IDEXX Bioanalytics (Columbia, MO, USA).

### 4.2. Production of the Multivalent Protein, QUAD 3.0

QUAD 3.0 protein consists of IL-13.E13K in the N-terminal end, CH_2_-CH_3_ regions of the human IgG1, and eA5 followed by a cysteine at the C-terminal end of the protein. QUAD 3.0 was produced in High Five^TM^ (Thermo Fischer, Waltham, MA) cells as described previously [46,85]. Briefly, the gene for QUAD 3.0 was cloned into pMIB V5 His A vector (Thermo Fischer, Waltham, MA, USA). The modified plasmid was introduced in High Five^TM^ cells using Cellfectin II reagent (Thermo Fischer, Waltham, MA, USA) as described by the manufacturer. Blasticidin was used to select the transfected insect cells. The protein was secreted and the QUAD 3.0-containing media, which was collected. QUAD 3.0 was purified on a HiTrap^®^ Protein G HP (GE, Boston, MA, USA) using fast protein liquid chromatography (FPLC) (GE, Boston, MA, USA) system.

### 4.3. Chemical Conjugation and Purification of Conjugates

QUAD 3.0 was designed to contain a cysteine, and therefore a thiol at the C-terminal end of the molecule. WP936 is a doxorubicin derivative-containing ready for reaction with thiol maleimide moiety [79,80]. QUAD 3.0 was conjugated with WP936, WP1737, and WP1244 under neutral conditions (0.1 M sodium phosphate, 0.15 M sodium chloride, pH 7.5) overnight at room temperature in a 1:3 molar ratio. Excess Dox was removed using 7K MWCO Zeba^TM^ desalting columns (Thermo Fischer, Waltham, MA, USA) to obtain a pure conjugate devoid of unconjugated Dox derivative. SDS-PAGE of the unconjugated and conjugated proteins was performed. Since the Dox derivatives are fluorescent, the SDS-PAGE gels were imaged on an Amersham AI600 RGB system (GE, Boston, MA, USA) at 570 nm to verify conjugation.

### 4.4. ELISA Binding Assays

First, 96-well ELISA plates were coated by incubation at 4 °C overnight with 100 μL of 1mg/mL of human recombinant EphA3, EphA2, EphB2, or IL-13RA2 (Sino Biological, Wayne, PA, USA). After the removal of excess coating protein, 2% milk/PBS was used to block nonspecific binding sites. QUAD 3.0 protein or QUAD 3.0 Dox conjugates diluted in 2% milk/PBS were added in various concentrations and incubated for 2 h at room temperature. After washing with PBS, HRP-labeled anti-human IgG1 (Jackson ImmunoResearch Inc., West Grove, PA, USA) was incubated for 1 h at room temperature. After the final wash, activated 2, 2′-azino-bis(3-ethylbenzothiazoline-6-sulphonic acid) (ABTS) (Sigma Aldrich, St. Louis, MO, USA) was added for detection and plates were read at 405 nm. Kd and Bmax were calculated by performing a nonlinear fit and a one site-specific binding model of data using GraphPad Prism version 7.04 for Windows, GraphPad Software, La Jolla California USA.

### 4.5. Drug to Vector (DAR) Ratio Determination

The measure of drug conjugation to ligand efficiency was established as follows: Absorbance of WP936 and QUAD 3.0-WP936 was measured at 280 and 488. The protein concentration of QUAD 3.0-WP936 was calculated by the following formula:

M = {[A280 of QUAD 3.0-WP936 − (A488 of QUAD 3.0-WP936 × CF)]/protein molar extinction coefficient},

where the correction factor (CF) is the A280/A488 of WP936 and the molar extinction coefficient of QUAD 3.0 is 69425.

The molar ratio (MR) was calculated the following formula:

MR = A488 of the conjugated protein/[8030 (molar extinction coefficient of WP936) × protein concentration (M)].

### 4.6. Cell Binding and Internalization of a QUAD 3.0-WP936 Conjugate

First, 1 × 10^4^ U-251 GBM cells were plated in each well of an 8-well chamber slide and allowed to adhere overnight. QUAD 3.0-WP936 conjugate was added in various amounts and incubated at 37 °C. Wells treated with PBS served as a control. After fixation with 10% buffered formalin, cells were permeabilized with PBS/1% BSA/0.1% Triton-X 100 for 10 min at 37 °C. QUAD 3.0 protein was detected by staining with Alexa-488-conjugated anti-human IgG (ThermoFisher Scientific, Waltham, MA) for 1 h at room temperature. Slides were washed with PBS and mounted with Fluoromount-G containing 4’,6-diamidino-2-phenylindole DAPI (Southern Biotech, Birmingham, AL, UK). WP936 fluoresces in the TRITC channel (excitation: 544 nm, emission: 570 nm). Images were captured on an Olympus IX70 microscope with a DP80 camera and compiled in CellSens software (Olympus, Waltham, MA, USA).

### 4.7. Cell Viability Assays

U-251, T98G, and BTCOE 4795 cells were plated in a 96-well plate and allowed to adhere overnight. Unconjugated Dox derivatives and QUAD 3.0-Dox conjugates in PBS/0.1% BSA were added to a final concentration of 100, 10, 1, 0.1, and 0.01 nM. After 72 h of drug exposure, cell viability was measured using the MTT assay (GoldBio, St. Louis, MO, USA) according to the manufacturer’s protocol. Absorbance was measured at 570 nm. Data were analyzed with GraphPad^®^ prism.

### 4.8. IC_50_ Value Determination

IC_50_ value is the concentration that corresponds to the response midway between the upper and lower estimated values. A four parametric logistic regression model was used. The concentrations of QUAD 3.0-Dox conjugates when the cell viability response corresponds to 50% of the control were calculated using the following mathematical Equation (1) (Sebaugh, J.L. Guidelines for accurate EC50/IC50 estimation. Pharm. Stat. 2011, 10, 128–134. doi:10.1002/pst.426):

(1) $$y=d+\frac{a-d}{1+{\left(\frac{x}{c}\right)}^{b}},$$

where,

‘*a*’ is the maximum value of y (response at 0 dose)
‘*b*’ is the slope factor or Hill Coefficient
‘*c*’ is the point of inflection (the point halfway between a and d, or IC_50_)
‘*d*’ is the minimum value of y (response at maximum dose).

### 4.9. In Vivo Toxicity

In order to investigate the toxicity of the QUAD 3.0-WP936 conjugate, 0.1, 0.5, and 1 μg of the conjugate in 5 μL (correspond to 167 nM, 833 nM and 1.7 μM, respectively) was intracranially injected in C57BL/6 mice. There were three mice per each group. Briefly, the heads of the mice were shaved prior to surgery. Once anesthetized with ketamine and xylazine, a scalp incision was made. Simulating a convection-enhanced delivery (CED) site in a rodent model [101], the intersection of coronal and sagittal sutures was identified as the bregma. Drug was stereotactically injected 1.0mm posterior to the bregma, 2.0 mm lateral to the sagittal suture, and 2.0 mm ventrally in the right hemisphere at a rate of 1 μL/min. The mice were monitored for pain, neurological symptoms, regular physical and grooming activities, and weighed daily for two weeks after the completion of the surgery. The studies were performed under IACUC protocol #A18-052 in compliance with the Animal Welfare Assurance with the Office for Laboratory Animal Welfare (A3045-01).

## Figures and Tables

**Table 1:** Kd and Bmax values of QUAD 3.0 and the QUAD 3.0 Dox conjugates binding to the EphA3, EphA2, EphB2, and IL-13RA2 receptors.

	EphA3		EphA2		EphB2		IL-13RA2	
	Kd	Bmax	Kd	Bmax	Kd	Bmax	Kd	Bmax
QUAD 3.0	0.25 nM	1.045	0.96 nM	0.67	2.2 nM	0.91	0.55 nM	0.26
QUAD 3.0-WP936	0.28 nM	1.1	1.9 nM	0.94	1.1 nM	0.93	2.6 nM	0.34
QUAD 3.0	0.14 nM	0.68	1.1 nM	0.60	0.53 nM	0.69	0.35 nM	0.72
QUAD 3.0-WP1737	0.09 nM	0.59	0.66 nM	0.20	1.6 nM	0.57	0.19 nM	0.35
QUAD 3.0-WP1244	0.13 nM	0.74	1.4 nM	0.10	13.9 nM	0.68	0.47 nM	0.36

**Table 2:** The IC_50_ values for QUAD 3.0 conjugates with Dox derivatives.

	QUAD 3.0-WP936	QUAD 3.0-WP1737	QUAD 3.0-WP1244
U-251	3.1 nM	2.4 nM	7.8 nM
BTCOE 4795	2.2 nM	3.7 nM	1.9 nM
T98G	1.1 nM	0.87 nM	2.5 nM
